# Knowledge of Adverse Drug Reactions Reporting among Doctors and Nurses in a Tertiary Care Hospital: A Descriptive Cross-sectional Study

**DOI:** 10.31729/jnma.5386

**Published:** 2021-01-31

**Authors:** Rekha Shah, Surya B. Parajuli, Suman Pokhrel

**Affiliations:** 1 Department of Pharmacology, Birat Medical College and Teaching Hospital, Biratnagar, Morang, Nepal; 2 Department of Community Medicine, Birat Medical College and Teaching Hospital, Morang, Nepal; 3 Department of Physiology, Birat Medical College and Teaching Hospital, Biratnagar, Morang, Nepal

**Keywords:** *adverse drug reaction*, *health care professionals*, *knowledge*

## Abstract

**Introduction:**

Doctors and nurses have a significant role in the detection of serious and unusual drug reactions. Effective implementation of an adverse drug reaction reporting system is required to ensure patient safety and quality care. This study's objective was to find the prevalence of good knowledge of adverse drug reaction reporting among the Doctors and nurses working in a tertiary care hospital.

**Methods:**

A descriptive cross-sectional study was conducted among doctors and nurses from 15 February 2020 to 15 July 2020 at Birat Medical College and Teaching Hospital. The convenience sampling method was used to select 192 study participants. A semi-structured questionnaire was used to know the knowledge concept of adverse drug reaction reporting. Ethical clearance was taken from IRC (PA-047/2076-77) of Birat Medical College and Teaching Hospital. Written informed consent was taken from each study participant. Collected data were entered in Microsoft Excel 2010 and analyzed by Statistical Package for the Social Sciences v23.

**Results:**

In total, 192 doctors and nurses, the questionnaires were distributed to 52 (27.1%) doctors and 140 (72.9%) nurses. The mean age of study participants was 28.14 years (SD ±4.5). To know the prevalence of knowledge, 15 knowledge related questions of adverse drug reaction had asked. The majority of doctors and nurses had good knowledge about adverse drug reaction reporting, 75% and 64%, respectively.

**Conclusions:**

Overall, doctors and nurses have had good knowledge of adverse drug reaction reporting. Data shows there is still more gap in training and experience on adverse drug reaction reporting systems.

## INTRODUCTION

According to WHO (1972), ADR is a response to a drug that is noxious and unintended and occurs at doses usually used in man for the prophylaxis, diagnosis, or therapy of disease, or the modifications of physiological function.^1^ ADR is a common cause of morbidity and mortality in both hospital and community settings.^2^ About 6-10% of all ADRs are reported worldwide. Among all healthcare professionals, doctors and nurses have a major vital role in detecting and reporting ADR.^3^ The reporting of ADR to pharmacovigilance centers were started in the mid-20th century after the thalidomide disaster.^4^

There are limited studies about knowledge in the Adverse Drug Reaction (ADR) reporting process in Nepal. This type of study also has not been done in our setting. Therefore, we are conducting this study in healthcare providers to sensitize them for active involvement in ADRs reporting activities, ultimately leading to a safer and more effective treatment for the patients.

This study aimed to find the prevalence of good knowledge of adverse drug reaction reporting among doctors and nurses working in Birat Medical College and Teaching Hospital.

## METHODS

A descriptive cross-sectional study was conducted from 15 February to 15 July 2020 at Birat Medical College and Teaching Hospital. The convenience sampling method was used to select 192 study participants. Ethical clearance was taken from IRC-PA-047/2076-77, Birat Medical College and Teaching Hospital, and written informed consent was taken from each participant. Convenience sampling was done. The sample size was calculated based on a study where the prevalence of good knowledge of ADRs reporting among doctors was 34.3%.^5^

The sample size was calculated as follow,

$$\begin{array}{ll} \text{n} & ={\text{Z}}^{\text{2}}\times (\text{p}\times \text{q)}/{\text{e}}^{\text{2}}\\ & =3.84\times 0.34\times 0.66/{\left(0.07\right)}^{\text{2}}\\ & =175. \end{array}$$

where,

n = minimum sample size
p = prevalence, 34.3%
q = 1-p
e = margin of error, 7%

Therefore, the calculated sample size was 175. Adding the non-response rate of 10%, the sample size was 192.

A total of 270 doctors and nurses were in the hospital at the time of data collection, comprising 75 doctors and 195 nurses. Data was collected from 192 health workers, among which 75 were doctors, and 140 were nurses. Pretesting was conducted among 19 doctors and nurses (10% of sample size) at Nobel Medical College Teaching Hospital. Appropriate modification of proforma done after pretesting. The questionnaire consisted of two sections; the first section included sociodemographic characteristics and the second section consisted of 15 questions regarding knowledge on ADR reporting. To know the good concept about ADRs reporting, knowledge related 15 questions had been asked to both the doctors and nurses. Each right answer was given the score of ‘1’ and each wrong answer was given the score of ‘0’.

Based on the individual mean score, knowledge was categorized as good and poor, i.e., score <8 (50%) participants’ knowledge was categorized as poor knowledge, and ≥8 was good knowledge. After explaining the objective of the study, doctors and nurses were enrolled. Those who were willing to take part in the study were included, and those who were not willing to participate in the study were excluded. The doctors and nurses were briefed about the rationale of the study and assured their privacy and confidentiality. Questionnaires were distributed along with informed consent in their respective department. According to their feasible time, the questionnaires have been filled. For this, the questionnaire sheet was kept with them for at least 45 minutes. After that, it has been recollected back on the same day. The questionnaires were left to those participants who were busy at that time and were collected the next day.

Data were entered into Microsoft Excel 2010 and analyzed by the statistical package for social sciences (SPSS) Version 23. The mean ± SD, percentage, and frequency were calculated.

## RESULTS

The majority were in the age group of 25 to 30 years, with a mean age of 28.14 (±4.5) years.

**Figure 1:**
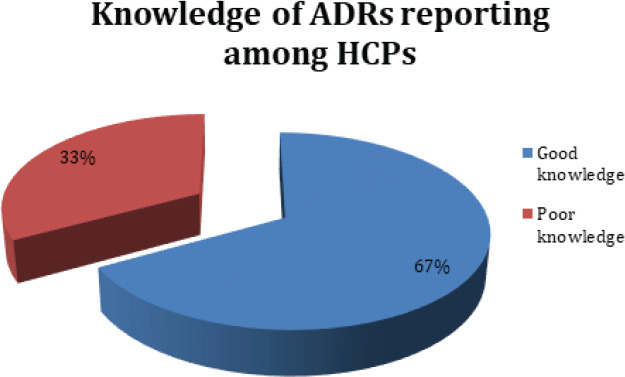
Distribution of good knowledge and poor knowledge of ADR among health care professionals.

**Figure 2:**
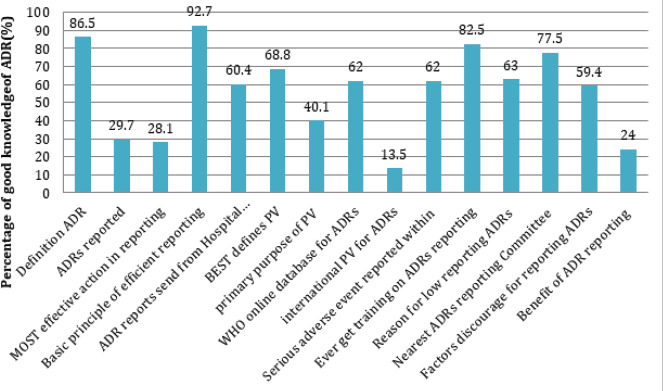
Correct response rates towards individual questions regarding knowledge of ADR by health care professionals.

The majority of healthcare professionals, 67%, have good, and 33% have poor knowledge about adverse drug reaction reporting. Participant's good knowledge regarding question answers of ADRs reporting is given in Figure 2.

*Pharmacovigilance = PV

Several items in the questionnaire were used to find out the prevalence of knowledge among doctors and nurses on ADR reporting. 166 (86.5%) respondents knew the definition of ADR, and 178 (92.7%) were able to know the basic principle of effective ADR reporting. Only 57 (29.7%) respondents knew how to report ADRs. Likewise, 132 (68.8%) knew the term pharmacovigilance and 77 (40.1%) understood its function. Moreover, a significant proportion of the respondents, 168 (82.5%) does not get any training on ADRs reporting, which may be the reason behind not knowing where and how to report ADRs 57 (29.7%). Only very minute participants, 25 (13.5%), were able to know the international pharmacovigilance center for ADR.

## DISCUSSION

In this study, both the healthcare workers showed adequate knowledge about the ADR reporting system doctor's 75% and 64% nurses. Under-reporting was one of the major problems in the study to limit the knowledge about ADR reporting systems. It was due to not knowing where and how to report ADRs. In Nepal, very few studies have been conducted on the ADR reporting program. Therefore, the present study was undertaken to know the knowledge of ADR reporting among doctors and nurses working at Birat Medical College and Teaching Hospital. Healthcare professionals play an important function in the spontaneous reporting of ADRs. ADRs reporting is a keystone of Pharmacovigilance centers throughout the world.^6^ All healthcare professionals should know which type of adverse effects to report, how and where to report will improve the adverse drug reaction reporting system.^7^ This study findings show that most nurses, 72.9%, followed by doctors, 27.1%, were enrolled. The proportion of enrollment of nurses and doctors were similar in other studies.^8,9^ We found the age group of participants was between 25-30 years in which most participants were graduate degree holders except staff nurses. A similar finding was reported in another study.^10^ We found the majority of doctors’ and nurses’ knowledge score on ADRs reporting was good, which is similar to other studies.^11,12^ Doctors has more knowledge score than nurses, which is similar to a study conducted in Ethiopia.^13^ The content of pharmacovigilance and ADRs reporting in the Nepali curriculum is not adequate.^14^ One of the basic worldwide problems seen in healthcare providers was underreporting in ADRs.^15,16^ The study done in south India showed that lack of remuneration was the main reason for the underreporting of ADR.^17^ Similar findings were found in our study. The studies of West Ethiopia and Nigeria showed a lack of knowledge as the main reason for underreporting ADRs, which is also true to our result, especially for nurses.^18,19^ Further studies suggested healthcare providers were unaware of the presence of regional pharmacovigilance centers. Contrary to our result, a study done in Sweden showed 60% reporting of ADR to an appropriate place.^20^ The reason behind the good reporting might be due to higher knowledge on ADRs reporting and better development of the reporting system. Most of our study participants had a complaint that they never attended any training on the ADRs reporting system. Lack of training and medical education regarding ADRs reporting was found to be the main reason behind poor knowledge of ADRs in several other studies.^20,21,22^ Hospital management and drug regulatory agencies have a great responsibility to improve the ADRs reporting system. For the good knowledge of ADRs reporting, the study done in India proved that the healthcare professionals who achieved training on education about ADRs reporting had sufficient knowledge of pharmacovigilance, and improvement on reporting systems ultimately led to good practice ADRs reporting. Interventional education and proper education at regular intervals increased alertness regarding ADRs reporting among healthcare providers.^21,22^ Developed countries like the UK, Sweden, Netherland, and France put the mandatory law for encountering ADRs to proper ADRs reporting center. Hence lead to 40-70% increased rates in the ADRs report.^23^ Therefore, continuous and regular education on up-to-date ADRs reporting systems and training programs should be arranged by the pharmacovigilance center. Even though healthcare professionals are encountering ADRs during their clinical practice, lack of reporting is a challenge for patient safety and quality. This reflects a need to enhance the doctors’ and nurses’ knowledge regarding ADR reporting.

## CONCLUSIONS

Despite doctors’ and nurses’ good knowledge about ADR reporting, continuing medical education on ADR reporting would help fill the know-do gap of ADR reporting among them.
